# Rapid Onset of Maternal Vocal Recognition in a Colonially Breeding Mammal, the Australian Sea Lion

**DOI:** 10.1371/journal.pone.0012195

**Published:** 2010-08-13

**Authors:** Benjamin J. Pitcher, Robert G. Harcourt, Isabelle Charrier

**Affiliations:** 1 Marine Mammal Research Group, Graduate School of the Environment, Macquarie University, Sydney, New South Wales, Australia; 2 Equipe Communications Acoustiques, Centre de Neurosciences Paris-Sud, UMR 8195, Université Paris Sud, Orsay, France; 3 Centre National de la Recherche Scientifique, Orsay, France; California Academy of Sciences, United States of America

## Abstract

**Background:**

In many gregarious mammals, mothers and offspring have developed the abilities to recognise each other using acoustic signals. Such capacity may develop at different rates after birth/parturition, varying between species and between the participants, i.e., mothers and young. Differences in selective pressures between species, and between mothers and offspring, are likely to drive the timing of the onset of mother-young recognition. We tested the ability of Australian sea lion mothers to identify their offspring by vocalisation, and examined the onset of this behaviour in these females. We hypothesise that a rapid onset of recognition may reflect an adaptation to a colonial lifestyle.

**Principal Findings:**

In a playback study maternal responses to own pup and non-filial vocalisations were compared at 12, 24 and every subsequent 24 hours until the females' first departure post-partum. Mothers showed a clear ability to recognise their pup's voice by 48 hours of age. At 24 hours mothers called more, at 48 hours they called sooner and at 72 hours they looked sooner in response to their own pup's vocalisations compared to those of non-filial pups.

**Conclusions:**

We demonstrate that Australian sea lion females can vocally identify offspring within two days of birth and before mothers leave to forage post-partum. We suggest that this rapid onset is a result of selection pressures imposed by a colonial lifestyle and may be seen in other colonial vertebrates. This is the first demonstration of the timing of the onset of maternal vocal recognition in a pinniped species.

## Introduction

In many species of gregarious birds and mammals, mothers and offspring have developed individual recognition abilities through acoustic channels [1], [2], [3], [4], [5], [6], [7], [8], [9], [10], [11], [12]. The timing of the development for such capacity after birth/parturition may vary among species and between the participants i.e., mothers and young [8], [13], [14]. Differences in selective pressures among species, and between mothers and offspring, are likely to drive the timing of mother-offspring recognition [15], [16], [17], [18], [19].

The need for individual recognition between parents and offspring is exacerbated by long periods of parental care, high risk of confusion due to high population density, and exclusive parental care of filial young (e.g., [10], [11], [12], [13]). Individual recognition between mothers and offspring can result in mutual benefits for both members of the dyad [20]. Mothers may increase their reproductive success by directing maternal care and resources to their own offspring; and young may limit their waste of energy in unsuccessful food solicitation from unrelated individuals that can potentially be highly aggressive to non-filial young [21], [22], [23], [24], [25].

Individual recognition systems, in particular parent-offspring recognition, can vary significantly between species in response to selection. The study of closely related species has shown how a single selection pressure, e.g. coloniality or offspring mobility, can markedly impact recognition abilities. Colonially breeding cliff swallow (*Hirundo pyrrhonota*) nestling vocalisations encoded 16 times more information than that of their relatives, the barn swallow (*H. rustica*) which are solitary [12]. An important consequence is that cliff swallow parents can vocally recognise their offspring, whereas barn swallow parents can not [26], [27]. Similarly, ungulate species whose young are mobile soon after birth (followers) exhibit mutual mother-offspring recognition (e.g., sheep, *Ovis aries*: [8], [28], [29]), whereas ‘hider’ species where offspring stay hidden (hiders), such as the fallow deer (*Dama dama*) show unidirectional recognition where only offspring can identify mothers [10]. While the influence of selective pressures on signal design and information encoding in individual recognition has been widely examined, the influence of selection on the timing of the onset of recognition has received little attention, particularly in mammals. In group-living species mother-offspring recognition should develop prior to the mixing of unrelated young, when other cues such as site-specific cues typically break down [30]. For instance, when nestlings fledge [31], [32], [33], when unrelated young begin to mix approaching weaning [24], or when the cost of provisioning unrelated young increases [23]. This situation is at its most extreme in gregarious, precocial, seasonally breeding species where the chances of encountering unrelated young is high [30]. Thus, in conjunction with the evolution of individually specific cues, the rapid onset of recognition is a way in which species may adapt to selection caused by a colonial lifestyle.

In mammals individual recognition processes can rely on different sensorial modalities such as olfaction, vision and audition. However the acoustic channel appears to be the most prevalent channel used in gregarious mammals [34]. Acoustic signals present a significant advantage in efficiency at both short and long range compared to other sensorial signals [34] as they allow for more reliable discrimination. Pinnipeds (seals, walrus, fur seals and sea lions) are good mammalian models for the study of acoustic communication since they use vocal signals in many social interactions including mother-pup recognition [15]. Mother-pup vocal recognition has been demonstrated in several species [15], [17], [35], [36], however the onset of this recognition process has been scarcely investigated in pups [13], [14], [17] and never in mothers [15].

**Figure 1:**
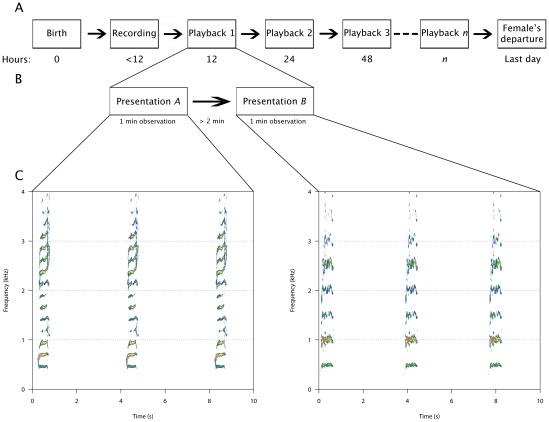
Experimental design. A. Females received the first playback within 12 hours of the birth of their pup, followed by another at 24 hours and every 24 hours until they departed the colony at the end of the perinatal attendance period. B. Each playback trial consisted of two presentations separated by a minimum of 2 minutes. During each presentation the responses of the female were recorded for 1 minute. The presentation order of own and non-filial pup's female attraction calls (FAC) was randomised. C. Presentations consisted of a single FAC repeated three times separated by 3 seconds of silence. Spectrograms were made using Seewave [48].

Australian sea lions (*Neophoca cinerea*) are endemic to the southern and south-western coasts of mainland Australia [37]. Females typically give birth to a single offspring and fast in the breeding colony for up to 14 days while suckling the young [38] during the perinatal attendance period (PAP). Following the PAP, females alternate between foraging trips and time ashore nursing (approximately 2 days each) for the duration of the 15 to 18 month lactation period [38]. Both mother and pup vocalisations have been shown to encode individual identity [39] and pups can use this information to identify their mother's voice [17], [36]. A recent study has shown that the Australian sea lion pup does not learn its mother's voice before being separated from her at the end of the PAP [17] suggesting that the mother drives reunions until the pup learns her call. However, there is no experimental evidence of vocal recognition of offspring by female Australian sea lions. In the present study we use a playback experiment (Figure 1) to test the hypothesis that Australian sea lion mothers can identify their offspring by voice, and we investigate the onset of this behaviour, suggesting that individual vocal recognition of pups develops before the mother leaves the colony at the end of the PAP. We hypothesise that a rapid onset would reflect an adaptation to colonial living and compensate for the slow onset of recognition behaviour seen in pups.

## Methods

### Ethics statement

This research was approved by the Department for Environment and Heritage, South Australia (Scientific permit E24934) and their Wildlife Ethics Committee, Approval 61/2005. All experimental procedures followed the Australian code of practice for the care and use of animals for scientific purposes.

### Study population

This study was conducted on Lewis Island, South Australia (34°57′19″ S, 136°01′53″ E) during June and July 2008. The colony had an estimated pup production of 131 individuals in 2007 [40]. At least twice daily throughout the study a thorough and systematic search of the island was conducted to identify any newborn pups, in addition opportunistic searches were conducted throughout the day. Immediately after a newborn pup was identified its female attraction calls (FAC) were recorded and the pup was marked with an individual symbol using hair dye (Clairol, Proctor & Gamble, Rydalmere, NSW, Australia).

### Vocalisation recording

Pup vocalisations were recorded using a BeyerDynamic M69TG (frequency response 50 Hz–16 kHz±2.5 dB, BeyerDynamic, Heilbronn, Germany) connected to a Marantz PMD 660 digital recorder (frequency response 16 kHz (−0.5 dB), dynamic range 80 dB). The microphone was enclosed in a Rycote Baby Ball Gag Windshield (Rycote Microphone Windshields, Stroud, United Kingdom), mounted on a 3m boom and held between 1–3 meters of the animal during recording. Recordings were made at 44.1 kHz sampling frequency with 16-bit resolution. FACs were re-recorded throughout the playback period to prevent pseudo-replication and account for potential changes in the pups' voice.

### Playback preparation

Playback series were prepared using Avisoft SAS Lab Pro (R. Specht, Avisoft Bioacoustics, Berlin, Germany) and Cool Edit Pro (Syntrillium Software Corporation, Phoenix, Arizona, USA). FACs were high-pass filtered at 200 Hz to remove wind and ocean noise. Filtering did not affect the FAC as there is little energy below 500 Hz [39]. Series of one FAC repeated three times, separated by three seconds, were constructed and normalised to ensure all series were broadcast at a consistent amplitude.

### Playback presentation

Females were presented with the FAC of their own pup and the FAC of a non-filial pup within 12 hours of birth, again at 24 hours post-birth and every 24 hours after that for the duration of the PAP, until the female left the colony on her first foraging trip post-partum. Playback series were presented using an Anchor Explorer Pro loudspeaker (30 W, frequency response 80 Hz–16 kHz±3dB, Anchor Audio, Torrance, California, USA) connected to the digital recorder. The loudspeaker was placed 3–4 metres from the tested female. To avoid any disturbance mothers and pups were not separated during playback sessions [17], instead we waited for the mother and pup to naturally move apart to a sufficient distance to induce the mother to search for the pup (i.e., >50 cm).

Each female received two series during a playback session; the FAC of her own pup and the FAC of a non-filial pup born to another female on the island during the same season. Series order was randomised and the observer was unaware of the type of FAC presented. To prevent habituation to the playback stimuli we played both different pup's and non-filial pup's FACs at each experimental presentation. Presentation of playback series was separated by at least 2 minutes.

### Response analysis

We recorded the responses of females for 60 seconds from the beginning of the playback series. The measured response behaviours on tested females were the latency to the first look to the playback calls, the latency to the first approach to the speaker, the latency to the first vocalisation and the total number of vocalisations given by the female. We analysed the data using two different methods. The first method consisted of performing a principal components analysis (PCA) of the raw behavioural data [41] for each test period (12h, 24h, 48h…Last day). The PCA included all the behaviours that were measured and was used to construct a composite score for the response to each playback type. The scores of the principal components with eigenvalues greater than 1 were compared using two-tailed Wilcoxon matched pair tests. The second method consisted of analysing each behavioural measurement separately and for each testing time period. Comparisons of behavioural responses obtained between non-filial and filial pups were performed using Wilcoxon matched pairs tests.

## Results

A total of 17 wild mother-pup pairs were studied during the perinatal attendance period. Thirteen females were followed for their entire PAP and had a mean attendance period of 8 days±1.47 s.d. (range 6–10). One female and her pup were not observed beyond 24 hours post birth while three had not departed by the end of our research trip. Because of this the following results report all individuals up to 96 hours post birth and we then present a “last day” giving the results for the 13 individuals tested on the last day of their PAP.

**Figure 2:**
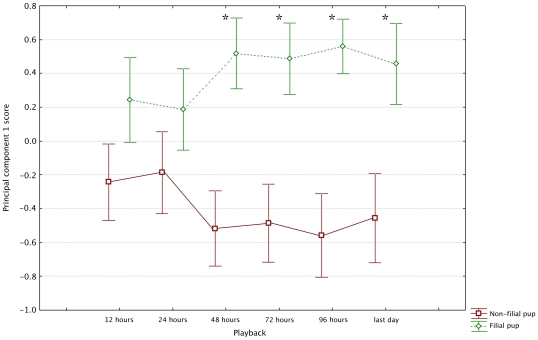
Comparison of composite response scores using principle component 1 of the PCA at each playback. From the first playback females show a trend to be more responsive to their filial pup's FAC. This difference becomes significant within 48 hours of birth. See table 1 for statistics, points = mean ± 1 S.E.M. * indicates significant difference.

The composite response scores obtained using PCA showed that females can reliably identify their offspring's voice within 48 hours of birth (Fig 2; Table 1). In all PCA, numbers of calls were positively correlated to PC1 and latencies negatively correlated to PC1. Higher values of PC1 correspond to a greater number of vocalisations given in response to the playback as well as shorter latencies to call, look and approach. Within 12 hours of birth, females showed a trend toward responding more toward the calls of their own offspring than a non-filial pup. However, within 48 hours of birth the females could reliably discriminate between their own pup's FAC and a non-filial pup's FAC.

**Table 1:** Summary of PCA.

	*Playback*	12 hours	24 hours	48 hours	72 hours	96 hours	Last day
	**N**	17	17	16	16	16	13
**PC1**	**Eigenvalue**	2.01	1.97	1.88	2.27	2.07	2.22
	**% of variance**	50.14	49.34	47.07	56.86	51.70	55.39
**Wilcoxon Test**	**Z**	1.86	1.78	3.35	3.07	2.84	3.11
	**p**	0.063	0.076	**0.00081**	**0.0022**	**0.0045**	**0.0019**

The composite response scores based on principal component 1, and the Wilcoxon matched pairs comparisons for each playback event. Significant differences in maternal responses between filial pup and non-filial FACs are highlighted in bold.

**Figure 3:**
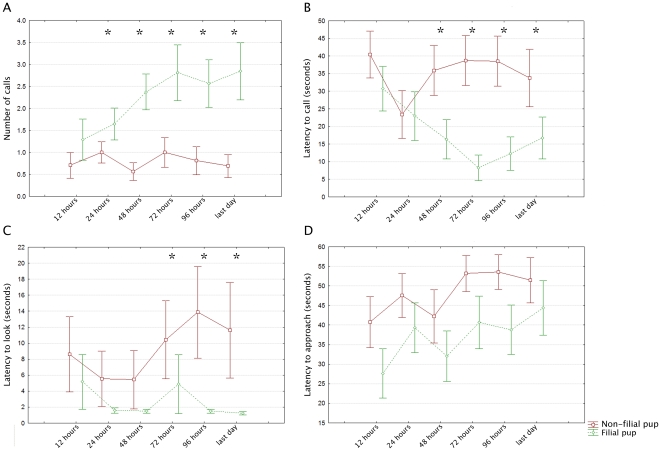
Comparisons of female responses within behavioural measures. A. Females call more in response to playback of their filial pup's and a non-filial pup's FACs within 24 hours of birth. See table 2, points = mean ± 1 S.E.M. * indicates significant difference. B. Within 48 hours of birth, females call sooner to the FAC of their filial pup than they do to the FAC of a non-filial pup. See table 2, points = mean ± 1 S.E.M. * indicates significant difference. C. By 72 hours after birth females look more rapidly to their filial pup's FAC than to a non-filial pup. See table 2, points = mean ± 1 S.E.M. * indicates significant difference. D. Females did not show a significant difference in their latencies to approach following playback of the FAC of their filial pup or a non-filial pup. See table 2, points = mean ± 1 S.E.M.

We obtained similar results when examining response behaviours individually, with more details being revealed about the development of the maternal response. Comparisons within each of the behavioural measures revealed a gradual onset and refinement of recognition over the first 72 hours of the pups' life. At 12 hours none of the behavioural measures showed significant differentiation between the females' responses to their pup's FAC or a non-filial pup's FAC. However, from 24 hours females gave more calls in response to their own pup's FAC than to the non-filial's (Table 2, Fig 3 A). Similarly, from 48 hours females were calling sooner (Table 2, Fig 3 B) and from 72 hours they were looking more rapidly to their own pup's FAC than to the non-filial pup's FAC (Table 2, Fig 3 C). Females did not show any differentiation between the calls in regards to the latency to approach (Table 2, Fig 3 D), potentially due to the limitations on their movements imposed by the presence of a mate-guarding male during the PAP.

**Table 2:** Summary of individual behaviour analysis.

Playback	12 hours		24 hours		48 hours		72 hours		96 hours		Last day	
N	17		17		16		16		16		13	
	Z	p	Z	p	Z	p	Z	p	Z	p	Z	p
**Number of calls**	−0.98	0.33	−2.24	**0.025**	−3.22	**0.001**	−3.22	**0.001**	−2.56	**0.010**	−2.95	**0.003**
**Latency to call**	−0.89	0.37	−0.12	0.91	−2.28	**0.023**	−2.87	**0.004**	−2.10	**0.036**	−2.36	**0.018**
**Latency to look**	−0.68	0.50	−0.93	0.35	−1.38	0.17	−1.94	**0.052**	−2.83	**0.005**	−2.82	**0.005**
**Latency to approach**	−1.57	0.12	−1.61	0.11	−1.01	0.31	−1.79	0.074	−1.40	0.16	−0.94	0.35

The Wilcoxon matched pairs comparisons within each behavioural measure for each playback event. Significant differences in maternal responses between filial pup's and non-filial pup's FACs are highlighted in bold.

## Discussion

Australian sea lion females can identify their offspring by their FAC alone within days of giving birth. This recognition ability begins to develop within 12 hours of birth with females showing a tendency to respond more to the calls of their own pup than to those of a non-filial pup. By 48 hours of age mothers can reliably identify their pup using vocal cues alone. The onset of this behaviour takes the form of a gradual increase in the selectivity of females' responses to pup vocalisations. Females begin by vocalising more in response to their own pup with 24 hours of birth. By 48 hours females vocalise more rapidly to their own pup and by 72 hours look faster in response to their own pups. This is the first experimental demonstration of the onset of maternal vocal recognition in a pinniped species.

The present study confirms that Australian sea lion mothers can identify their offspring by voice. Acoustic analysis of pup vocalisations has shown that they produce individually stereotyped vocalisations, and that this vocal signature involves the fundamental frequency, the energy spectrum, and both the frequency and amplitude modulations of the calls [39]. While further playback studies are necessary to identify the specific components of the vocal signature mothers use to identify their offspring our results show that this discrimination is possible within 48 hours of birth. It is likely that Australian sea lions use a combination of sensory signals during individual recognition, i.e. auditory, olfactory and visual [17], [ 36], [ 42, Pitcher et al. unpublished data] and that when used in concert the onset of maternal recognition is more rapid, potentially within 12 hours of birth.

The timing of the onset of maternal recognition of pup vocalisations is dependant on both the sender and the receiver of the signals [30]; in this case the pup and the mother respectively. As the sender of the signal, the pup must produce not only the vocalisations, but there must be a sufficient level of individual distinctiveness in those vocalisations to allow for discrimination [43]. Because of the potential benefits of being recognised by mothers, pup vocalisations are likely to be under selection for individual distinctiveness [43], but must still develop a level of consistency to allow for recognition. Pup vocalisations may go through a period of rapid development soon after birth, before becoming more stable. The fine acoustic structure of the FACs of subantarctic fur seal (*Arctocephalus tropicalis*) pups change over the duration of the maternal care period (from 1–210 days of age). However, this change is gradual enough that mothers continue to learn and recognise the ‘new’ call [44]. The results of our study suggest that pup vocalisations have developed both a sufficient level of distinctiveness and stability to be recognised by 48 hours after birth. As the receiver, in order to recognise her pup's vocalisation, the mother must have the ability to differentiate between vocalisations. It is likely that the perceptual and cognitive abilities to differentiate between individuals develop as a pup, before two months of age when they first recognise their own mother's vocalisations [17]. Therefore, the timing of the onset of recognition seen in this study is a result of the amount of exposure that is necessary for a mother to form a model of her pup's vocalisation, or the time taken for the pup to develop an individually distinctive vocalisation, or most probably a combination of the two.

While in this species a mother's ability to recognise her offspring is likely to be mutually beneficial to both mothers and pups, allowing mothers to direct resources to filial young, it is probable that parent-offspring conflict [20] plays a role in mediating the behaviour of mothers and young and affects the timing of onset. Parent-offspring conflict theory predicts that parents should maximise their lifetime reproductive success by controlling investment in individual young, while young should attempt to maximize their share of parental care and resources [20]. Trivers [20] suggests that throughout the period of parental investment mothers will initiate contact more in the early stages of investment, while offspring initiate contact more in the latter stages. Such a change in behaviour during the period of postnatal parental care reflects the changes in costs and benefits to each party. Our results show that Australian sea lion mothers develop the ability to identify their offspring's vocalisations more rapidly than offspring develop the ability to recognise their mother's vocalisation [this paper, 17]. Indeed, pups do not show vocal recognition abilities before the end of the PAP and instead develop these abilities at some time before two months of age. Mothers act to control investment in offspring by directing reunions through strong and early established individual recognition behaviour. Similar evidence for parent-offspring conflict is seen in another pinniped species, the northern fur seal [*Callorhinus ursinus*: 4]. Northern fur seal mothers appear to control their parental investment by being more discerning in vocal recognition than their pups. Mothers are less likely to respond to the vocalisation of a non-filial pup, than a pup is to respond to another female other than its mother [4]. For pups, the cost of incorrectly rejecting a call is greater than responding to false alarms, and the cost of incorrectly rejecting a call is greater for pups than mothers [4]. This is consistent with different selection pressures acting on mothers and pups, as predicted by parent-offspring conflict theory [20]. In Australian sea lions, the rapid onset of maternal recognition of pups may contribute to the reduction of selection pressures on pups as mother-pup reunions are driven by mothers, particularly in the early stages of maternal care. This low pressure may combine with low colony density and natal site fidelity [17]. In contrast mothers are likely to be under high selective pressure through parent-offspring conflict and the costs of misdirected parental care. Thus the difference in the timing of the onset of recognition seen between mothers and pups is likely to be a result of the different selection pressures facing the members of the dyad.

In group-living species mother-offspring recognition should develop prior to mixing of unrelated young [23], [24], [30], [31], [32], [33]. However, very few studies have experimentally examined the onset of maternal vocal recognition of offspring in mammals. Most previous studies have focused on domesticated species and humans. In sheep (*Ovis aries*), ewes can identify their lambs voice within 24 hours of birth [8], and goats (*Capra capra*) can identify their kids using a combination of vocal and visual cues within 4 hours of birth [18], [19]. In humans (*Homo sapiens*), mothers appear to discriminate the cries of their own child from another's within 48 hours of birth [45], [46]. Otariids are typically gregarious, seasonal breeders with precocial young [47]. Not only must mothers be able to identify their pups before separating from them during foraging trips, but there is also a high risk of confusion during the perinatal period when multiple pups are born in close proximity [22]. The rapid onset of recognition seen in Australian sea lion mothers conforms to the expectation that recognition is present before the separation of mothers and pups, and shows that mothers can recognise their offspring soon after birth, well before the end of the perinatal period.

In conclusion, it appears that as an adaptation to a colonial breeding system, Australian sea lion mothers can identify their offspring using vocal cues alone within 48 hours post-partum, and it is likely that when used in concert with other senses this recognition is present within 12 hours of birth. While further study is necessary to identify which components of the pup vocal signature mothers are using for identification, it is clear that this signature is a reliable indicator of identity from an early age. We suggest that rapid onset of recognition behaviour may be a response to the selection pressures imposed by colonial living, and is likely to be seen in other colonial vertebrates. Further investigation of the onset of recognition across a wider array of species would allow for a greater understanding of the development of mammalian communication and recognition systems.
